# Arsenic Hyperaccumulation Strategies: An Overview

**DOI:** 10.3389/fcell.2017.00067

**Published:** 2017-07-18

**Authors:** Zahra Souri, Naser Karimi, Luisa M. Sandalio

**Affiliations:** ^1^Laboratory of Plant Physiology, Department of Biology, Faculty of Science, Razi University Kermanshah, Iran; ^2^Laboratory of Oxygen and Nitrogen Species Signalling Under Plant Stress Conditions, Department of Biochemistry and Molecular and Cellular Biology of Plants, Estación Experimental del Zaidín, Consejo Superior de Investigaciones Científicas Granada, Spain

**Keywords:** arsenic, glutathione, hyperaccumulators, nitric oxide, phytochelatins, phytoremediation, reactive oxygen species

## Abstract

Arsenic (As) pollution, which is on the increase around the world, poses a growing threat to the environment. Phytoremediation, an important green technology, uses different strategies, including As uptake, transport, translocation, and detoxification, to remediate this metalloid. Arsenic hyperaccumulator plants have developed various strategies to accumulate and tolerate high concentrations of As. In these plants, the formation of AsIII complexes with GSH and phytochelatins and their transport into root and shoot vacuoles constitute important mechanisms for coping with As stress. The oxidative stress induced by reactive oxygen species (ROS) production is one of the principal toxic effects of As; moreover, the strong antioxidative defenses in hyperaccumulator plants could constitute an important As detoxification strategy. On the other hand, nitric oxide activates antioxidant enzyme and phytochelatins biosynthesis which enhances As stress tolerance in plants. Although several studies have focused on transcription, metabolomics, and proteomic changes in plants induced by As, the mechanisms involved in As transport, translocation, and detoxification in hyperaccumulator plants need to be studied in greater depth. This review updates recent progress made in the study of As uptake, translocation, chelation, and detoxification in As hyperaccumulator plants.

## Introduction

The metalloid arsenic (As), a ubiquitous contaminant widely found in organisms and the environment, has had severe, chronic and epidemic effects on human, plant, and animal health in South-East Asia (Clemens and Ma, 2016). Arsenic exists in different states (−III, 0, +III, and +V), mainly as arsenate (AsV) and arsenite (AsIII), and exhibits a wide range of solubility depending on the ionic environment and pH. AsV, a phosphate chemical analog, enters the plant system through phosphate transporters, causing imbalances in phosphate supply (Finnegan and Chen, 2012). AsIII presents in reducing environments such as flooded paddy soils at pH < 8 in general, is more toxic and mobile than AsV (Kumar et al., 2015). Once in the cell, AsV interferes with the phosphate-dependent metabolism by replacing phosphate in phosphorolytic reactions, including ATP synthesis, thus causing toxicity in plant cells. However, AsIII toxicity is mainly due to its tendency to react with thiol (−SH) groups of enzymes and proteins containing cysteine residues which disturb their structure and function (Finnegan and Chen, 2012; Hasanuzzaman et al., 2015).

In nature, few plant species are capable of accumulating or detoxifying extraordinarily high levels of As. Hyperaccumulator plants have adopted an array of approaches to facilitate the elimination, accumulation, and hyperaccumulation of toxic metals (Islam et al., 2015; Ghori et al., 2016). The ability of plants to accumulate and tolerate As can be exploited to develop phytoremediation technologies to improve food safety. A number of species have been identified as As hyperaccumulators, most of which belong to the *Pteridaceae* family (Xie et al., 2009). A new specie recently considered as As hyperacumulator is *Isatis cappadocica* a plant found in Iran whose As tolerance strategy involves increasing thiol synthesis and As chelation with glutathione and PCs (Karimi et al., 2009). By contrast, the fern *Pteris vittata* is equipped with efficient systems for AsV/AsIII uptake, translocation to shoots, and As sequestration in vacuoles (Xie et al., 2009; Danh et al., 2014). The stem and leaves of the *P. vittata* fern showed no significant changes in tissue or cell structure caused by As, which was accumulated along the walls of vascular stem bundles and, to a lesser extent, in roots (Sridhar et al., 2011). Energy-dispersive x-ray microanalysis showed that As was mainly located in the epidermal cell vacuoles of *P. vittata* fronds (Lombi et al., 2002).

This review updates the progress made in the study of the mechanisms involved in As transport, translocation and detoxification, as well as the role played by reactive oxygen species (ROS) and nitric oxide (NO) in As detoxification in As hyperaccumulator plants.

### Phytoremediation

Phytoremediation, a green approach using plants to remediate toxic compounds, is a cost-effective, socially acceptable, and environmentally friendly technology for soil, and groundwater clean-up (Jiang et al., 2015). This technology uses metal-tolerant and hyperaccumulator plants which require high growth rates, tolerance to large heavy metal concentrations, and the capacity to accumulate high levels of heavy metals in their above-ground parts (over 100–1,000 mg/kg depending on the metal involved; Ghori et al., 2016). Plant hyperaccumulators require a root-to-shoot heavy metal concentration ratio (translocation factor, TF) of over 1 and a root-to-soil heavy metal concentration ratio (bioconcentration factor, BC) also exceeding 1 (Ghori et al., 2016). Based on these criteria it can be concluded that *I. cappadocica* is an As hyperaccumulator due to its capacity to tolerate and accumulate As, exceeding the 1,000 mg/kg threshold for As, which is at least one order of magnitude higher than in other species at the same As contaminated area, without showing any As toxicity symptoms (Karimi et al., 2009). Phytoremediation has been broadly categorized into different process such as phytoextraction/phytoaccumulation, phytostabilization, phytodegradation/phytotransformation, rhizofiltration, rhizodegradation, phytovolatilization, and phytorestauration (Fayiga and Saha, 2016). Phytoextraction has been extensively studied as a clean-up solution for soils contaminated by metal pollutants through their absorption by roots and subsequent translocation to plant shoots. Certain higher plant species, with specific genetic and physiological potential, which can accumulate, translocate, and tolerate very high metal concentrations in their tissues without showing toxicity, are useful for phytoextraction purposes (Danh et al., 2014). Such naturally occurring plants, called metal hyperaccumulators, could be suitable for phytoremediation. While several plant species are capable of hyperaccumulating and detoxifying extraordinarily high levels of heavy metal, only a few plant species (*P. vittata, Pteris criteca, Pteris longifolia, Pteris umbrosa, Pitrogram macalomelanos*, and *I. cappadocica*) are known to be As hyperaccumulators (Kumar et al., 2015). Two of these species, *P. vittata* (Xie et al., 2009) and *I. cappadocica* (Karimi et al., 2009) are regarded as efficient models to decipher the mechanisms involved in As hyperaccumulation and tolerance.

## Mechanisms involved in As hyperaccumulation

### Arsenic uptake, transport, and translocation

**Figure 1:**
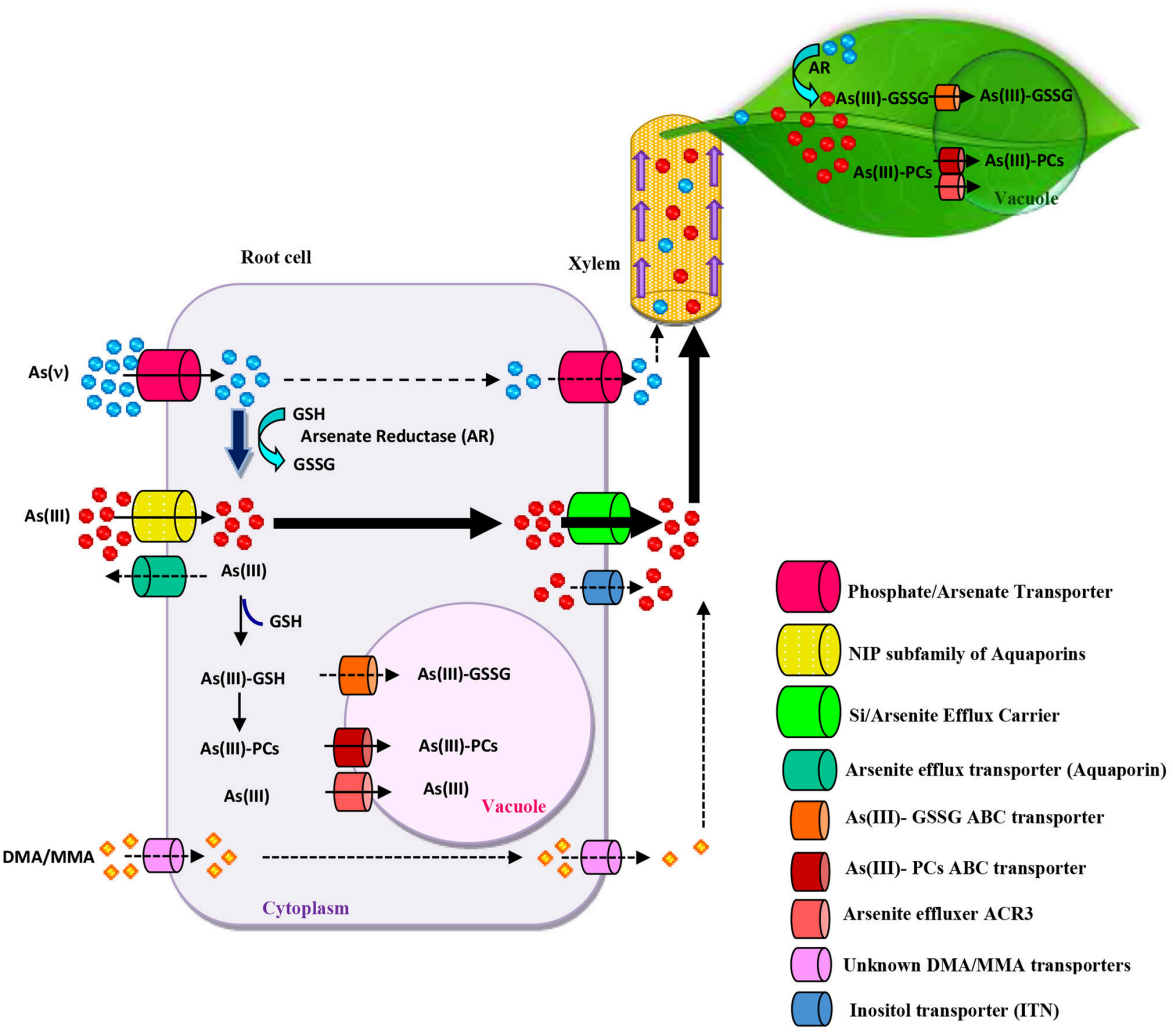
Overview of Arsenic (As) uptake, transport, translocation, and detoxification in plants. Arsenate (AsV) uptake can occur via phosphate transporters. AsV reduction occurs in root cells before xylem loading and transportation to shoots. Arsenate reductase (AR) reduces AsV to arsenite (AsIII) by using glutathione (GSH) as a reductant. AsIII uptake occurs via nodulin 26-like intrinsic (NIP) aquaglyceroporin channels. Arsenic methylated species (DMA/MMA) uptake is carried out by unknown transporters or by NIP. Phytochelatins (PCs) and GSH coordinate with AsIII to form a variety of complexes which are sequestered in vacuoles by ABC-type transporters. In *Pteris vitatta*, As(III) can also be transported to the vacuole by Arsenical Compound Resistance3 (ACR3) effluxer. As loading to the xylem can be mediated by the Si/Arsenite efflux transporters or inositol transporters (INT). The considerable capacity for As root-to-shoot translocation and vacuolar sequestration in shoots ensures high As deposition levels in the above-ground part.

The rate of As uptake and accumulation by plants depends on factors such as soil type, As speciation, plant species and uptake mechanisms (Zhao et al., 2009). The characteristics of the soil, such as pH, water content, organic substances, and As content, regulate As bioavailability to roots (Finnegan and Chen, 2012; Huang et al., 2012). As speciation in soil through different As forms (AsV, AsIII and methylated As) is another factor, which can significantly affect As uptake by plants. Plant roots selectively take up specific As forms via distinct pathways and transporters (Gupta et al., 2011; Farooq et al., 2016). AsV is taken up via high-affinity Pi transporters (Figure 1) following Michaelis-Menten kinetics in higher plants including *P. vittata* and *I. cappadocica* (Su et al., 2008; Karimi and Souri, 2015). Evidence for this has been provided by physiological and radiotracer 73 AsV uptake studies which show competitive inhibition of AsV uptake by Pi (Abedin et al., 2002; Karimi et al., 2009) and the isolation of AsV-resistant *Arabidopsis thaliana* mutants defective in or over-expressing Pi transporters (Shin et al., 2004; Catarecha et al., 2007). Pi uptake is highly regulated in plants, and similar mechanisms may also regulate AsV uptake and translocation (Sun et al., 2012).

Different types of transporters have been reported to be involved in AsIII uptake. One belonging to the plant aquaporin family, nodulin 26-like intrinsic proteins (NIPs) (Zhao et al., 2009; Chen et al., 2016). NIP1;1, NIP1;2, NIP3;1; NIP5;1; NIP6;1; and NIP7;1, are involved in arsenous acid (the neutral chemical form of AsIII) uptake and transport in Arabidopsis roots (Bienert et al., 2008; Xu et al., 2015). In rice plants, OsNIP3;3 and HvNIP1;2 have also been characterized as arsenous acid-permeable NIPs (Katsuhara et al., 2014). Furthermore, two silicic acid transporters, Lsi1 (also called NIP2;1) and Lsi2, facilitate arsenous acid transport in rice roots (Ma et al., 2008; Singh et al., 2015). However, a competitive inhibition study suggests that neither glycerol nor silicic acid affect AsIII uptake in *P. vittata*, indicating that the AsIII uptake system in this and probably other As hyperaccumulators, differs from that reported in rice (He et al., 2016). In *P. vittata*, a new aquaporin tonoplast intrinsic protein (TIP), called PvTIP4, has been reported to be involved in AsIII uptake (He et al., 2016). AsIII transporters in *P. vittata* roots show a much higher affinity than those in rice roots, which explains *P. vittata'*s extraordinary As uptake capacity (Chen et al., 2016). Transporters responsible for inositol uptake (INT) in the phloem in Arabidopsis also transport arsenic, and the disruption of AtINT2 or AtINT4 in these plants led to a reduction of arsenic concentration in phloem, silique, and seed (Duan et al., 2015). However, whether there are similar transporters responsible for As transport in hyperaccumulators plants is still unknown. After entering plants, volatilization or efflux of As in roots can reduce As translocation to shoot in As-tolerant/nonhyperaccumulator plants. By contrast, translocation of As to shoots in hyperaccumulators are highly efficient, and efflux levels are insignificant (Su et al., 2008; Chen et al., 2016). In root cells, As is either converted to less toxic organic forms or is transported to vacuoles as AsIII or as AsIII-glutathione/phytochelatin complexes (Figure 1; Kumar et al., 2015). This mechanism occurs efficiently in the roots of hypertolerant/non-hyperaccumulator plants, thus preventing As translocation to shoots (Figure 1). AsV reduction to AsIII has been reported to occur efficiently in hyperaccumulator plants. Previous studies have identified AsIII as the predominant form of As transported in the xylem sap from root to shoot, regardless of whether AsV or AsIII is supplied to the plants (Raab et al., 2005; Su et al., 2008). The extremely efficient As translocation in As hyperaccumulators could probably due to the effective reduction of AsV to AsIII in roots; to the high AsIII efflux from cortical cells to the xylem; limited thiol compound complexation of AsIII and sequestration in root vacuoles; in addition to minimal AsIII efflux from roots to the external medium (Su et al., 2008; Karimi et al., 2009; Zhao et al., 2009; Indriolo et al., 2010). As hyperaccumulators, such as *I. cappadocica* and *P. vittata*, therefore accumulate approximately 60–80% of As in shoots, with an As shoot to root ratio of over 1 (Karimi et al., 2013; Chen et al., 2016), while only 5–10% of total As is found in the shoots of non-accumulating species such as the *P. tremula* fern (Caille et al., 2005), Arabidopsis (Isayenkov and Maathuis, 2008), and rice (Ma et al., 2008).

The capacity to load large amounts of As in the xylem is an important feature of As hyperaccumulators (Figure 1), although the mechanisms involved are poorly understood. In *P. vittata*, both high- and low-affinity systems are involved in this process (Wang et al., 2011). In rice, AsIII and Si share the same pathways for both root uptake and xylem-mediated loading processes through OsLsi2 (Ma et al., 2008). Additionally, the Pi transport pathway may be involved in the long-distance translocation of As with OsPht1;8 overexpression markedly increasing AsV translocation from roots to shoots and the AsV/AsIII ratio in the xylem sap of rice (Wu et al., 2011). In sunflower plants, no As-sulfhydryl complexes, such as As-GSH and As-PC, were found in the sap exudate, suggesting that AsIII and AsV are sap-mobile forms of As in these plants (Raab et al., 2005). Plants, including the hyperaccumulator *P. vittata*, can also uptake methylated As species, which are more cytotoxic; interestingly, methylated species, such as monomethylarsonic acid (MMA) and dimethylarsinate (DMA), are more mobile than inorganic As species in the xylem and phloem; however, the key methylated As transport regulators in these tissues have not yet been clearly identified (Huang et al., 2008; Li et al., 2009). In rice roots, Lsi1 is involved in the influx of methylated As species (Ma et al., 2006; Li et al., 2009). Although thiol complexation in rice roots is an important step in MMA metabolism, this is not the case for DMA (Mishra et al., 2017).

### Arsenic detoxification

Reduction of AsV to AsIII is accepted as the first step in the detoxification of As in plant tissues by promoting AsIII efflux to the external medium. The reduction of AsV to AsIII occurs enzymatically via the arsenate reductase (AR) pathway using GSH (Figure 1) (Finnegan and Chen, 2012). AR genes, such as *AtHAC1/ATQ1* (Arabidopsis) (Chao et al., 2014; Sánchez-Bermejo et al., 2014), OsHAC1;1 and OsHAC1;2 (rice) (Shi et al., 2016), *HlAsr* (*Holcus lanatus*) (Bleeker et al., 2006) have been cloned and characterized. Recent evidence showed that canonical ACR2 does not play a significant role in arsenate reduction (Liu et al., 2012; Chao et al., 2014). AR activity observed in *P. vittata* was at least 7-fold higher than that in As-sensitive plant species such as *Oryza sativa* and *A. thaliana* (Duan et al., 2005; Danh et al., 2014). The high expression levels of AR and vacuolar AsIII transporters in shoots may explain the special ability of *P. vittata* to hyper-tolerate and hyper-accumulate As compared to other As-sensitive plants (Song et al., 2010).

Another important As detoxification strategy in hyper-accumulating plants is the synthesis of glutathione (GSH) and phytochelatines (PCs) which produces complexes with As that facilitate its transport into the vacuoles in shoots (Figure 1; Karimi et al., 2009; Zhao et al., 2009). The tripeptide GSH (Glu-Cys-Gly) is synthesized by γ-glutamyl cysteine synthetase (γ-ECS) and GSH synthetase (GS). GSH can bind to AsIII and is also a key metabolite in the cellular redox balance (Figure 1) (Jozefczak et al., 2012; Islam et al., 2015). GSH is the precursor of PCs, whose rate of accumulation is usually increased by γ-ECS or GS overexpression under As exposure (Zhao et al., 2009). PCs are a family of small enzymatically synthesized peptides composed of oligomers of GSH with the structure (γ-Glu-Cys)n-Gly, with n ranging from 2 to 11 (Batista et al., 2014). Other evidence shows that PC complexation of AsIII is an important mechanism in both constitutive and adaptive tolerance to As in As non-hyperaccumulating plants (Gupta et al., 2011). Transgenic plants overexpressing genes regulating cysteine, GSH and PC biosynthesis show greater As detoxification capacity (Tripathi et al., 2007; Wojas et al., 2008). However, further studies have shown that PCs appear to play a minor role in direct As detoxification in *P. vittata* due to the extremely low molar ratio of PCs to As observed in this species (Zhao et al., 2009; Jedynak et al., 2012). On the other hand, the hypertoleration and accumulation of larger amounts of above-ground As in *I. cappadocica* were achieved by PC complexation (>50%) which is regarded as a constitutive tolerance mechanism (Karimi et al., 2009). These findings suggest that PCs play a crucial role in As detoxification; although do not contribute significantly to As tolerance in certain hypertolerants *(H. lanatus* and *Silene paradoxa*) and hyperaccumulators (*P. vittata* and *P. cretica*) plant species (Raab et al., 2007; Arnetoli et al., 2008). Sequestration of the AsIII-PCs complexes in vacuoles is an important step in As detoxification metabolism. In *A. thaliana*, two ABC transporters AtABCC1 and AtABCC2, which have been located in the vacuole, play an important role in As resistance (Figure 1) (Song et al., 2010). In rice, OsABCC1 transports AsIII-PCs across the tonoplast; its overexpression in yeast and Arabidopsis increases As tolerance, while knockout mutants are hypersensitive to the metalloid (Song et al., 2014). A PDR-like protein, a member of the ABC transporter G family, was upregulated by As stress in *P. vitatta* (Shen et al., 2014). Indriolo et al. (2010) showed that As hyperaccumulation in *P. vittata* is associated with the AsIII effluxer Arsenical Compound Resistance3 (ACR3), which is localized to the vacuolar membrane in gametophytes but it has not been identified in angiosperms.

## Role of ROS and NO in As toxicity and tolerance

### Reactive oxygen species

Exposure of plants to As stress increases ROS accumulation through the disruption of electron transport chains (ETC) in mitochondria and chloroplasts, glycolate oxidase activation, antioxidant inactivation, and GSH depletion (Gupta et al., 2013; Fayiga and Saha, 2016). Several studies have shown a positive correlation between greater antioxidant capacity, mainly in above-ground parts, and metal and As tolerance in hyperaccumulator plants (Visioli and Marmiroli, 2013; Kumar et al., 2015; Fayiga and Saha, 2016; Karimi and Souri, 2016). In *P. vittata*, the large GSH pool helps to minimize As-induced oxidative stress and enhances As tolerance (Singh et al., 2006). Moreover, ROS, particularly H_2_O_2_, play an important role as signaling molecules which participate in the complex network regulating cell responses to As (Sharma, 2012; Thao et al., 2015). It has recently been reported that ROS produced by NADPH oxidase C (NOXC) in Arabidopsis plants can regulate the uptake and translocation of As and various nutrients, although the mechanism involved is not fully understood (Gupta et al., 2017). H_2_O_2_ is also implicated inthe activation of several Mitogen-activated protein kinase (MAPKs) under As stress (Huang et al., 2012). MAPK transduce and amplify the signals through a cascade of reversible phosphorylation processes. Huang et al. (2012) have found 11 MAPK kinases (MAPKKKs), one MAPK and 10 phosphatases (PP2C) genes significantly upregulated in rice treated with AsV. Some of these MAPKs have been involved in the regulation of the sulfate assimilation pathway (Ahsan et al., 2008; Norton et al., 2008) and the regulation of ethylene and jasmonic acid signaling pathways in response to metals (Opdenakker et al., 2012).

### Nitric oxide

NO, a hydrophobic diffusible gaseous molecule, plays an important signaling role in physiological processes and responses to heavy metal stresses (Lamattina et al., 2003; Farnese et al., 2013; Singh et al., 2015; Fancy et al., 2017). It regulates different biological processes in plants by directly modifying proteins via post-translational modifications (PTMs), leading to *S*-nitrosylation, nitration, and nitrosylation, and indirectly by regulating gene transcription (Astier and Lindermayr, 2012; Romero-Puertas et al., 2013; Romero-Puertas and Sandalio, 2016a; Fancy et al., 2017). Several studies have demonstrated that exogenous NO attenuate oxidative stresses imposed by As (Farnese et al., 2013; Singh et al., 2015). NO can act as a ROS scavenger and antioxidant system inducer, although the molecular mechanism involved is not fully understood (Farnese et al., 2013; Singh et al., 2016). NO-dependent S-nitrosylation can regulate the H_2_O_2_ level by controlling both the antioxidant defense system (CAT, SOD, APX, and peroxiredoxins) and ROS producing enzymes (glycolate oxidase and NADPH oxidases) (Romero-Puertas and Sandalio, 2016a,b). Peroxynitrite (ONOO^−^) can also nitrate and regulate antioxidant defenses (APX, SODs; Romero-Puertas and Sandalio, 2016a). It has been reported that metal nitrosylation can also affect the capacity of PCs to chelate Cd (De Michele et al., 2009; Locato et al., 2016). In addition, NO could regulate As accumulation by down-regulating *OsLis1* and *OsLis2* in rice (Singh et al., 2016) and by up-regulating ABC transporters (Hussain et al., 2016). In addition, NO stimulates sulfate uptake and PC biosynthesis (Farnese et al., 2013; Singh et al., 2016) and also plays an important role in metal and As signaling through activation of MAPKs (Hahn and Harter, 2009; Ye et al., 2013).

## Conclusions

In nature, a small number of plant species capable of accumulating and detoxifying extraordinarily high levels of As have been identified. These hyperaccumulator plants have developed coordinated strategies to As uptake, transport and translocation to shoots, As chelation with GSH and phytochelatins and its efficient transport to vacuoles, in addition to an efficient antioxidant system regulated by ROS and NO. Major advances have been made in relation to non-accumulating As-tolerant plant species in terms of As uptake and transport. Nevertheless, As transport, translocation and detoxification in hyperaccumulator plants such as *I. cappadocica* require more in-depth study in order to understand how As accumulation functions in these plants, which can be used for phytoremediation purposes. Finally, much study has deservedly been devoted to the role played by ROS and NO in the regulation of As uptake and translocation, which have been emerging as key players in metal uptake and homeostasis.

## Author contributions

ZS: Wrote most of the manuscript. NK: An expert in this field, made valuable suggestions, and supervised the work of ZS. LS: Conceived, designed, and coordinated the review and also wrote some of the text.

### Conflict of interest statement

The authors declare that the research was conducted in the absence of any commercial or financial relationships that could be construed as a potential conflict of interest.
